# Food-Seeking Behavior Is Mediated by Fos-Expressing Neuronal Ensembles Formed at First Learning in Rats

**DOI:** 10.1523/ENEURO.0373-20.2021

**Published:** 2021-04-05

**Authors:** Richard Quintana-Feliciano, Christina Gobin, Louisa Kane, Bo Sortman, Samantha Rakela, Ariana Genovese, Brendan Tunstall, Daniele Caprioli, Sergio D. Iñiguez, Brandon L. Warren

**Affiliations:** 1 Department of Pharmacodynamics, University of Florida, Gainesville, FL 32610; 2 Behavioral Neuroscience Branch, Intramural Research Program/National Institute on Drug Abuse/National Institutes of Health/Department of Health and Human Services, Baltimore, MD 21224; 3 Santa Lucia Foundation (Istituto di Ricovero e Cura a Carattere Scientifico Fondazione Santa Lucia), Rome 00185, Italy; 4 Department of Physiology and Pharmacology, Sapienza University of Rome, Rome, Italy; 5 Department of Psychology, The University of Texas at El Paso, El Paso, TX 79968; 6 Department of Pharmacology, University of Tennessee Health Science Center, Memphis, TN 38163

**Keywords:** acquisition, daun02, food seeking, operant conditioning, PFC

## Abstract

Neuronal ensembles in the infralimbic cortex (IL) develop after prolonged food self-administration training. However, rats demonstrate evidence of learning the food self-administration response as early as day 1, with responding quickly increasing to asymptotic levels. Since the contribution of individual brain regions to task performance shifts over the course of training, it remains unclear whether IL ensembles are gradually formed and refined over the course of extensive operant training, or whether functionally-relevant ensembles might be recruited and formed as early as the initial acquisition of food self-administration behavior. Here, we aimed to determine the role of IL ensembles at the earliest possible point after demonstrable learning of a response-outcome association. We first allowed rats to lever press for palatable food pellets and stopped training rats once their behavior evidenced the response-outcome association (learners). We compared their food-seeking behavior and neuronal activation (Fos protein expression) to similarly trained rats that did not form this association (non-learners). Learners had greater food-seeking behavior and neuronal activation within the medial prefrontal cortex (mPFC), suggesting that mPFC subregions might encode initial food self-administration memories. To test the functional relevance of mPFC Fos-expressing ensembles to subsequent food seeking, we tested region-wide inactivation of the IL using muscimol+baclofen and neuronal ensemble-specific ablation using the Daun02 inactivation procedure. Both region-wide inactivation and ensemble-specific inactivation of the IL significantly decreased food seeking. These data suggest that IL neuronal ensembles form during initial learning of food self-administration behavior, and furthermore, that these ensembles play a functional role in food seeking.

## Significance Statement

Neuronal ensembles within the infralimbic cortex (IL) play a causal role in mediating established food self-administration and food seeking. Here, we conducted region-wide and neuronal ensemble-specific inactivation within the IL to determine whether IL neuronal ensembles are involved in initial acquisition of food self-administration behavior. We demonstrate that neuronal ensembles within the IL control initial learning of food self-administration behavior.

## Introduction

Food self-administration involves learned associations between stimuli, response, and outcome, and is a useful behavior in studying the development and persistence of food-seeking. The corticostriatal pathway has been implicated in controlling food-seeking. Opposing roles of the prelimbic cortex (PL) and infralimbic cortex (IL) have been proposed in the initiation and suppression of appetitive seeking, respectively (Rhodes and Killcross, 2004; Ishikawa et al., 2008a; LaLumiere et al., 2012; Sangha et al., 2014; Gourley and Taylor, 2016; Trask et al., 2017). However, disparate findings, wherein some studies demonstrate opposing roles of these regions or no effect, suggest a more nuanced role of the dorsal versus ventral subregions of the medial prefrontal cortex (mPFC) in mediating appetitive behaviors (Burgos-Robles et al., 2013; Keistler et al., 2015; Moorman and Aston-Jones, 2015; Gentry and Roesch, 2018; Caballero et al., 2019; Riaz et al., 2019). These findings highlight the limitation of global inactivation compared with targeting behaviorally-relevant neuronal ensembles (Hebb, 1949; Cruz et al., 2015). Indeed, single neurons have been shown to contribute to appetitive behavior (Burgos-Robles et al., 2013; Moorman and Aston-Jones, 2015), and the existence of separate but intermingled neuronal ensembles within the IL have been demonstrated to selectively encode opposing behaviors (Suto et al., 2016; Warren et al., 2016). Given that learned associations between reinforcers and predictive stimuli are encoded with such a high degree of specificity, techniques with sufficient mechanistic resolution are necessary (Warren et al., 2017). The recently developed Daun02 chemogenetic procedure in *Fos-LacZ* transgenic rats allows the selective manipulation of activity-defined subpopulations of neurons (Cruz et al., 2013).

Fos expression is a common proxy marker of neuronal activity and is commonly used to identify neuronal ensembles (Cruz et al., 2015). In *Fos-LacZ* transgenic rats, strong neuronal activity induces co-expression of β-galactosidase (β-gal) and Fos protein only in strongly activated neurons. Microinfusing the prodrug Daun02 during maximal β-gal protein coexpression (∼90 min after the start of behavioral testing) enables β-gal to hydrolyze Daun02 into Daunorubicin, effectively precipitating apoptosis exclusively in strongly activated neuronal ensembles (Cruz et al., 2013; Koya et al., 2016).

Using Daun02 inactivation to selectively ablate behaviorally relevant Fos-expressing neuronal ensembles, researchers have found that intermingling neuronal ensembles within the IL modulate different reward-specific memories associated with food-seeking and extinction of food-seeking in rats that underwent extensive food self-administration training (Warren et al., 2016). Furthermore, distinct neuronal ensembles within the IL mediate seeking for a food versus cocaine reward when rats are trained extensively to self-administer both reinforcers (Kane, 2020). Similarly, others have demonstrated that the IL plays a role in self-administration in overtrained rats (Coutureau and Killcross, 2003; Killcross and Coutureau, 2003). Together, these studies suggest that IL ensembles specific to established reward-related memories develop with extensive training. However, it is unknown whether IL neuronal ensembles are recruited during the initial acquisition of self-administration behavior, often observable during the very first day of food self-administration training.

The contribution of individual brain regions to task performance can shift as an animal transitions from the initial acquisition or rudimentary learning of task completion, to the maximally efficient performance of a “mastered” task (Kupferschmidt et al., 2017; Bradfield et al., 2020). We hypothesized that even in the earliest stages of food self-administration learning, neuronal ensembles are formed in the IL and are necessary to guide food-seeking behavior. We designed the following experiments to test this hypothesis.

First, we developed a novel procedure to study the food-seeking behavior of rats at the earliest point of demonstrable food-self administration learning (learners) with rats matched in terms of training history but that did not acquire food-self-administration (non-learners). We demonstrated that learners, contrasted with non-learners, demonstrate food-seeking behavior and that this was coupled with quantifiable elevations of Fos protein expression in the dorsal and ventral mPFC. Next, to test the role of the IL brain region in food-seeking in learners, we inactivated the region broadly with the use of a GABA_A_+GABA_B_ agonist cocktail (muscimol+baclofen), expected to interfere with the function of all cells in the region. Following-up this study, we applied the more refined approach of Daun02 inactivation to determine whether within this region inactivating a small percentage of Fos expressing cells (<5%; a neuronal ensemble) might be sufficient to similarly disrupt the food-self-administration memory and thereby disrupt food-seeking behavior.

## Materials and Methods

### Subjects

We used male and female Sprague Dawley wild-type rats (*n* = 84) as well as male and female *Fos-lacZ* transgenic rats (*n* = 45), weighing 175–400 g at the beginning of the experiments. Rats were pair-housed by sex, maintained on a reverse 12/12 h light/dark cycle (lights off at 10 A.M.), and given *ad libitum* access to food and water. All animal procedures were approved by the relevant Institutional Animal Care and Use Committees. A total of six wild-type and four *Fos-LacZ* transgenic rats were excluded for misplaced cannulas, and one wild-type rat was excluded from experiment 1 for poor labeling.

### Surgery

We anesthetized rats with isoflurane and injected buprenorphine (0.03 mg/kg, s.c.) and meloxicam (2 mg/kg, s.c.) for 3 d after surgery to relieve pain. Rats were given a 5-d recovery period before behavioral testing.

### Intracranial cannula implantation

We implanted permanent guide cannulas (23-gauge, Plastics One) bilaterally 1 mm above the IL. The nose bar was set at −3.3 mm, and the coordinates for the IL were anteroposterior: +3.0, mediolateral: ±1.5, and dorsoventral: −3.8 (10° angle). We fixed cannulas to the rat’s skull with dental cement and jeweler’s screws. We used the above coordinates based on pilot and previous studies (Bossert et al., 2011; Warren et al., 2016, 2019).

### Intracranial injections

We performed intracranial injections using a syringe pump (Kent Scientific) and 10 μl Hamilton syringes that were attached via polyethylene-50 tubing to 30-gauge injectors (RWD) that extended 1 mm beyond the guide cannula. We infused 0.5 μl over 1 min and left the injectors in place for an additional 1 min before removal.

### Drugs

In experiment 2, we injected the GABA_A_ (muscimol, 0.03 nmol/0.5 ul/side, MilliporeSigma) and GABA_B_ (baclofen, 0.3 nmol/0.5 ul/side, Alfa Aesar) agonists into the IL. These drugs were dissolved in sterile saline. The chosen concentration is based on previous studies (McFarland and Kalivas, 2001; Bossert et al., 2011; Warren et al., 2016). Daun02 was obtained from Medchem Express and dissolved (2 μg/0.5 μl/side) in vehicle solution containing 5% DMSO, 6% Tween 80, and 89% 0.01 m PBS. We chose the dose of Daun02 based on previous studies (Koya et al., 2009; Bossert et al., 2011; Cruz et al., 2014a; Warren et al., 2016).

### Apparatus

Rats were habituated, trained, and tested in Med Associates self-administration chambers; each equipped with a house light, fan, retractable active and inactive levers, a cue light positioned above the active lever and a central food port. The house light and fan remained on throughout the session. Pressing the active lever resulted in activation of the cue light directly above the active lever, and delivery of a palatable food pellet into the food port. The food port was fitted with an infrared sensor to record head entries. Pressing the inactive lever had no programmed consequences.

### Fos and NeuN immunofluorescence

We washed coronal brain sections (40 μm) from experiment 1 in 1× PBS, blocked with 3% normal goat serum (NGS) in 1× PBS with 0.25% Triton X-100 (PBS-Tx), and incubated 24 h at 4°C with anti-Fos antibody (1:5000 dilution; Cell Signaling Technology catalog # 5348, RRID: AB_10557109) in blocking solution. We then washed sections in 1× PBS, and incubated them with Alexa fluor 488 conjugated goat anti-rabbit secondary antibody (1:500 dilution; Thermo Fisher Scientific catalog #A-11 008, RRID: AB_143165) and Alexa Fluor 568 conjugated anti-mouse secondary antibody (1:500 dilution; Thermo Fisher Scientific catalog #A-1104, RRID: AB_141371). Fluorescent images of immunoreactive cells in the IL, PL, and NAc were captured using a Keyence BZ-X810 microscope at 20× magnification with BZ-X Analyzer software. Cell profiler was used to automatically count total number of cells labeled with NeuN from two sections (bilateral) per rat (three images per rat). Number of Fos-positive nuclei in these images were manually counted by observers blind to the test conditions (interrater reliability: Pearson’s correlate *r *=* *0.93). We averaged the counts so that each rat was an *n* of 1 for each brain area. The percentage of total average Fos counts out of total average NeuN counts for each rat was the dependent measure for Fos expression for each region.

### Self-administration training for food

Each experiment consisted of 1–5 d of self-administration training in which we placed rats into operant chambers (described above) for 2 h/d and trained them to press the active lever for food on an FR1 schedule of reinforcement with a 20 s timeout. Each 2 h session was broken up into two 1 h sessions, with a 10 min break between sessions. Based on preliminary experiments, we selected acquisition criteria of at least 50 active lever presses and >75% responding on the active lever to operationalize acquisition of food self-administration behavior. We chose this threshold based on previous experiments that included more training sessions (Warren et al., 2016; Kane, 2020) and based on earlier studies that applied similar acquisition criteria (Mitchell et al., 2005; Haluk and Wickman, 2010; Taylor et al., 2010; Derman and Ferrario, 2020). Therefore, we used this threshold to categorize a rat as a learner or non-learner. The duration of the self-administration phase was variable for individual rats because on reaching criteria (acquisition of lever pressing for food), we terminated self-administration training for learner rats. We also stopped training an equal number of non-learner rats to match learner and non-learner rats for handling and exposure to the apparatus. We also measured head entries to confirm that rats were collecting the food pellets earned during the session.

### Muscimol±baclofen inactivation

In experiment 2, we microinfused muscimol+baclofen into the IL 10 min before a 15-min recall test. During the recall test (1 d after the last training session), presses on the active lever resulted in activation of the cue light but no reward delivery. Ninety min after the start of the test, we deeply anesthetized rats with isoflurane and transcardially perfused them with 1× PBS followed by 4% paraformaldehyde (PFA) solution. We postfixed brains for 2 h at 4°C, cryopreserved them in 30% sucrose and kept them frozen at −80°C until sectioning.

### Daun02 inactivation

In experiment 3, on induction day, 1 d after the last training session, we exposed the rats to a brief (15 min) food seeking test wherein presses on the active lever resulted in activation of the cue light but no pellet delivery. The purpose of this test was to induce Fos expression related to acquisition of the food reward learned during the self-administration phase. Because this test occurred shortly after rats met criteria or acquired the food seeking behavior, the Fos expression exhibited during the induction test is likely to be associated with the initial food acquisition memory. Ninety min following the start of the recall test, we bilaterally microinfused rats with either vehicle or Daun02 into the IL to inactivate neuronal ensembles associated with the food acquisition memory. Two days later, we retested rats in a second 15-min recall test to assess the effects of Daun02 or vehicle on recall of the food acquisition memory. Ninety min after the start of the test, we deeply anesthetized rats with isoflurane and transcardially perfused them with 1× PBS followed by 4% PFA solution. We postfixed brains for 2 h at 4°C, cryopreserved them in 30% sucrose and kept them frozen at −80°C until sectioning.

### Experimental design and statistical analysis

#### Experiment 1: Fos expression in the mPFC in learners versus non-learners following a food recall test

The purpose of experiment 1 was to determine whether exposure to cues previously associated with food self-administration induced different patterns of Fos expression within subregions of the mPFC (PL and IL) for rats categorized as learners or non-learners. This experiment was a single-factor between-subjects design with two groups (learners vs non-learners). Male and female rats were given the opportunity to self-administer palatable food pellets for 2 h over the course of 3 d. Rats reaching the minimum criteria of 50 active lever presses and >75% responses on the active versus inactive lever during one session were considered learners, while the rest were deemed non-learners. We based these criteria on observations from previous experiments and from previous studies that applied acquisition criteria (Mitchell et al., 2005; Haluk and Wickman, 2010; Taylor et al., 2010; Derman and Ferrario, 2020). Once a rat reached 50 lever presses in a single session, they were excluded from further training sessions. In order to match learner and non-learner rats for exposure to training sessions, we also excluded an equal number of non-learners each day. In this experiment, four male rats became learners and five became non-learners; three female rats became learners and four became non-learners. The day following the last self-administration session, we assessed rats on non-reinforced active lever pressing during a 15-min recall test. The purpose of this test was to reactivate the memory associated with food self-administration (Fanous et al., 2012; Cruz et al., 2014b; Koya et al., 2016; Warren et al., 2016, 2019; Caprioli et al., 2017). We transcardially perfused rats 75 min after this recall test to target peak Fos expression associated with this task. Greater Fos expression in a particular region for the learners compared with the non-learners was expected to indicate involvement of that region in mediating initiation of food seeking. Because rats are tested in this final test on their non-reinforced behavioral responding, Fos expression is not expected to be confounded by reinforcing properties of the food pellet. Rather, Fos expression is expected to be induced by memory of the initial food reward formed during acquisition in the food self-administration period. We processed the brains for immunohistochemistry as described above.

#### Experiment 2: effect of muscimol±baclofen in the IL during recall of food self-administration memories

The purpose of experiment 2 was to assess the effect of region-wide inactivation of the IL on the recall of food self-administration memories. We used a 2 × 2 factorial design: group (learners vs non-learners) × drug (vehicle, muscimol+baclofen). Twenty-four hours after the last day of food self-administration, we microinfused muscimol+baclofen or vehicle into the IL 10 min before testing rats on non-reinforced behavioral responding during the 15-min recall test. We perfused rats 75 min after the end of the recall test and harvested tissue for cannulae placement. In this experiment, 20 male rats became learners and 14 became non-learners; 15 female rats became learners and 10 became non-learners.

#### Experiment 3: effect of Daun02 inactivation of Fos expressing neuronal ensembles in the IL during recall of food self-administration memories

The purpose of experiment 3 was to assess the effect of inactivating Fos-expressing neuronal ensembles within the IL on the recall of food self-administration memories. We used a 2 × 2 factorial design: group (learners vs non-learners) × drug (vehicle, Daun02). Twenty-four hours after the last day of food self-administration, we tested rats on non-reinforced behavioral responding during a 15-min test. We microinfused Daun02 or vehicle into the IL 75 min following the end of this test to selectively ablate neurons that were activated during the induction session. Two days later, we tested rats on non-reinforced behavioral responding during a 15-min recall test (identical to the induction session). We perfused rats 75 min after the end of the recall test and harvested tissue for cannulae placement. In this experiment, 16 male rats became learners and 11 became non-learners; eight female rats became learners and six became non-learners.

### Statistical analysis

We analyzed all data using GraphPad Prism (version 8.31) software, setting the α level at 0.05 for all statistical analyses used. We used two-way ANOVAs or unpaired *t* tests to analyze all behavioral and immunohistochemical data when appropriate. We followed significant main effects and interactions detected in ANOVA with Holm–Sidak *post hoc* analyses. We found no sex differences in test-day lever pressing in any experiment (*p* > 0.05) and so collapsed data obtained from male and female rats for all analyses. For detailed statistical information, see Table 1.

**Table 1:** Statistical table

Figure	Data structure	Type of test	Factors	Statistical data
1*D*	Normal distribution	Unpaired Student’s *t* test	Group (non-learner, learner)	*t*_(13)_ = 2.71, *p* = 0.02, Cohen’s *d* =1.4
1*E*	Normal distribution	Unpaired Student’s *t* test	Group (non-learner, learner)	*t*_(13)_ = 2.69, *p* = 0.02, Cohen’s *d* =1.4
2*C*	Normal distribution	Two-way ANOVA (Holm–Sidak *post hoc* analyses)	Group (non-learner, learner) × preassigned drug (vehicle, muscimol+baclofen)	**Main effect of group** (*F*_(1,49)_ = 84.6, *p* < 0.0001, η_p_² = 0.64); no main effect of preassigned drug condition (*F*_(1,49)_ = 84.6, *p* = 0.92, η_p_² < 0.001); and no group × preassigned drug interaction (*F*_(1,49)_ < 0.001, *p* = 0.99, η_p_² < 0.001) Vehicle: learner vs non-learner *p* < 0.0001 muscimol+baclofen: learner vs non-learner *p* < 0.0001
2*D*	Normal distribution	Two-way ANOVA	Group (non-learner, learner) × drug (vehicle, muscimol+baclofen)	**Main effect of group** (*F*_(1,49)_ = 60.0, *p* < 0.0001, η_p_² = 0.55); **main effect of drug condition** (*F*_(1,49)_ = 10.9, *p* = 0.002, η_p_² = 0.18); and **group × drug interaction** (*F*_(1,49)_ = 12.14, *p* = 0.001, η_p_² = 0.2)
2*E*	Normal distribution	Two-way ANOVA	Group (non-learner, learner) × drug (vehicle, muscimol+baclofen)	**Main effect of group** (*F*_(1,49)_ = 7.9, *p* = 0.007, η_p_² = 0.14); no main effect of drug condition (*F*_(1,49)_ = 0.26, *p* = 0.61, η_p_² = 0.005); and no group × drug interaction (*F*_(1,49)_ = 0.0005, *p* = 0.98, η_p_² < 0.001)
2*F*	Normal distribution	Two-way ANOVA	Group (non-learner, learner) × drug (vehicle, muscimol+baclofen)	**Main effect of group** (*F*_(1,49)_ = 20.3, *p* < 0.0001, η_p_² = 0.29); no main effect of drug condition (*F*_(1,49)_ = 2.5, *p* = 0.12, η_p_² = 0.048); and a **group × drug interaction** (*F*_(1,49)_ = 4.25, *p* = 0.045, η_p_² = 0.08)
3*C*	Normal distribution	Two-way ANOVA (Holm–Sidak *post hoc* analyses)	Group (non-learner, learner) × preassigned drug (vehicle, daun02)	**Main effect of group** (*F*_(1,37)_ = 38.7, *p* < 0.0001, η_p_² = 0.51); no main effect of preassigned drug condition (*F*_(1,37)_ = 0.09, *p* = 0.76, η_p_² = 0.0.002); and no significant group × preassigned drug interaction (*F*_(1,37)_ = 0.09, *p* = 0.76, η_p_² = 0.002) vehicle: learner vs non-learner *p* = 0.0004 muscimol+baclofen: learner vs non-learner *p* < 0.0001
3*D*	Normal distribution	Two-way ANOVA (Holm–Sidak *post hoc* analyses)	Group (non-learner, learner) × drug (vehicle, daun02)	**Main effect of group** (*F*_(1,37)_ = 24.1, *p* < 0.0001, η_p_² = 0.39); no main effect of drug condition (*F*_(1,37)_ = 0.46, *p* = 0.5, η_p_² = 0.01); and **a significant group × drug interaction** (*F*_(1,37)_ = 4.5, *p* = 0.042, η_p_² = 0.11) non-learner: vehicle vs daun02 *p* = 0.37 **learner: vehicle vs daun02 *p* = 0.048**
3*E*	Normal distribution	Two-way ANOVA	Group (non-learner, learner) × drug (vehicle, daun02)	**Main effect of group** (*F*_(1,37)_ = 8.4, *p* = 0.006, η_p_² = 0.19); no main effect of drug condition (*F*_(1,37)_ = 0.001, *p* = 0.97, η_p_² < 0.0001); and no group × drug interaction (*F*_(1,37)_ = 0.04, *p* = 0.83, η_p_² = 0.001)
3*F*	Normal distribution	Two-way ANOVA	Group (non-learner, learner) × drug (vehicle, daun02)	**Main effect of group** (*F*_(1,37)_ = 7.9, *p* = 0.001, η_p_² = 0.18); no main effect of drug condition (*F*_(1,37)_ = 0.07, *p* = 0.79, η_p_² = 0.002); and no group × drug interaction (*F*_(1,37)_ = 0.9, *p* = 0.34, η_p_² = 0.02)

Detailed statistical analysis for each figure. Significant effects shown in bold.

## Results

### Experiment 1: Fos expression in the mPFC subregions in learners versus non-learners following a food recall test

**Figure 1.**
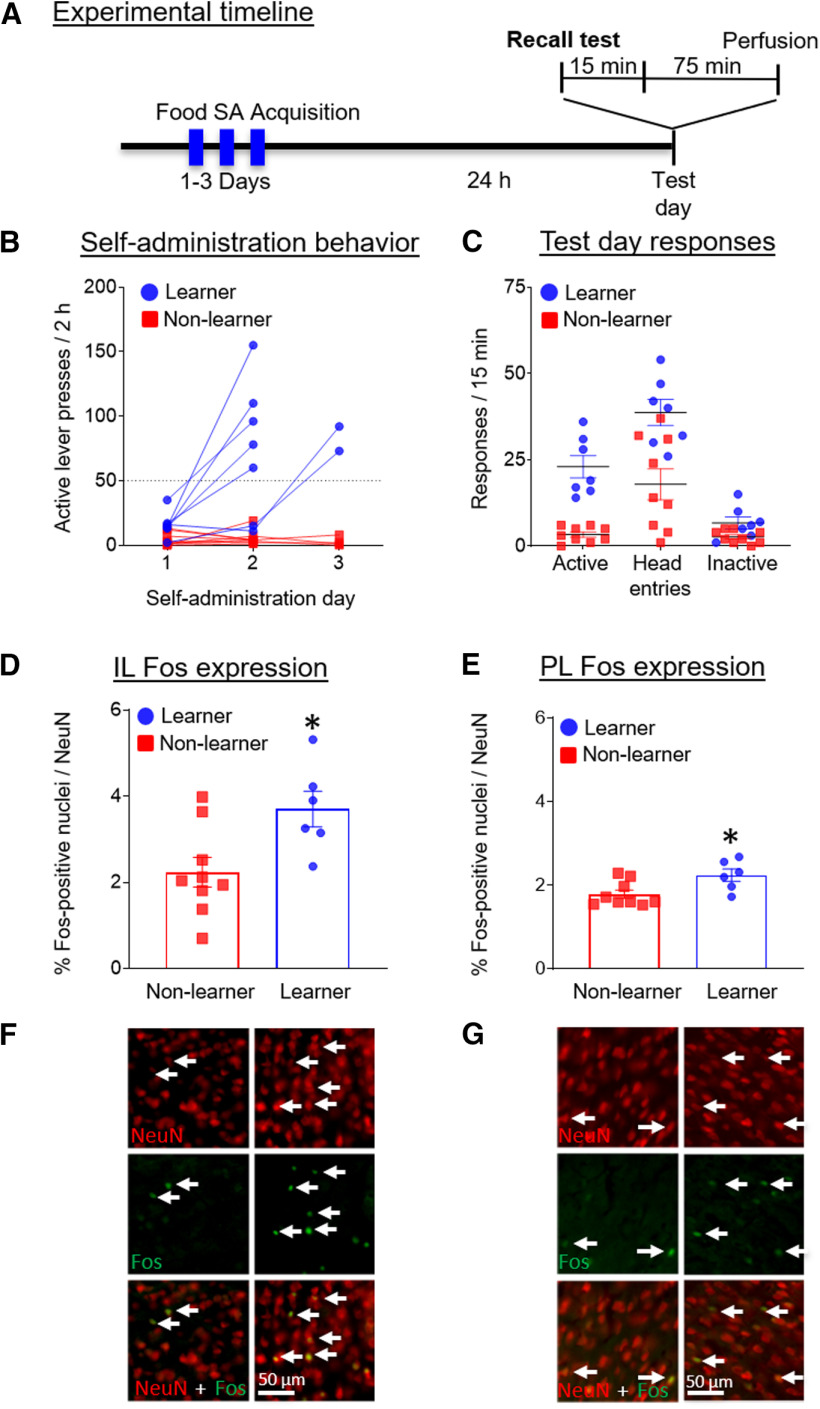
Experiment 1: Fos expression in mPFC subregions in learners versus non-learners following a food recall test. ***A***, Timeline showing the behavioral procedure. We trained rats to self-administer (SA) food for between 1 and 3 d in 2-h daily sessions, until rats self-segregated into learners and non-learners. On testing day, we allowed the rats to lever press for food for 15 min under non-reinforced conditions and perfused them 75 min later to measure Fos expression. ***B***, Number of active lever presses for food across training sessions. ***C***, Number of active lever presses, head entries, and inactive lever presses during the 15-min test day session. ***D***, Percentage of Fos-positive nuclei/NeuN-positive neurons in the IL per mm^2^ for rats on test day before perfusions. ***E***, Percentage of Fos-positive nuclei/NeuN-positive neurons in the PL per mm^2^ for rats on test day before perfusions. ***F***, Representative labeling within the IL. ***G***, Representative labeling within the PL. Scale bar: 50 μm. Data are presented as mean ± SEM (*n* = 6–9 per group); *p < 0.05.

The timeline for experiment 1 is shown in Figure 1*A*. Figure 1*B* shows the number of active lever presses during the self-administration phase. Food acquisition was operationalized as meeting the minimum criteria of 50 active lever presses in a single session, with >75% responding on the active versus inactive lever. In accordance with these criteria, rats self-segregated into learners or non-learners across the 3 d of training (Fig. 1*B*). Learners also demonstrated more head entries during the terminal session, indicating that the rats were indeed collecting the food pellets in a timely manner, further demonstrating that the rats had learned the response-outcome association. Figure 1*C* shows non-reinforced behavioral responding (active lever presses, inactive lever presses, and head entries into the foodport) during a 15-min recall test. We do not include statistical tests of the differences in behavioral responses between learners and non-learners because they have non-overlapping distributions as a result of their selection criteria.

We found no significant differences in Fos expression between male and female rats and therefore combined their data for analysis. We used separate unpaired *t* tests to analyze group differences in Fos expression in the IL (Fig. 1*D*) and the PL (Fig. 1*E*) following the test recall sessions. Learners showed significantly greater Fos expression compared with non-learners in the IL (*t*_(13)_ = 2.71, *p* = 0.02, Cohen’s *d* =1.4) and in the PL (*t*_(13)_ = 2.69, *p* = 0.02, Cohen’s *d* =1.4).

### Experiment 2: effect of muscimol±baclofen in the IL during recall of food self-administration

**Figure 2.**
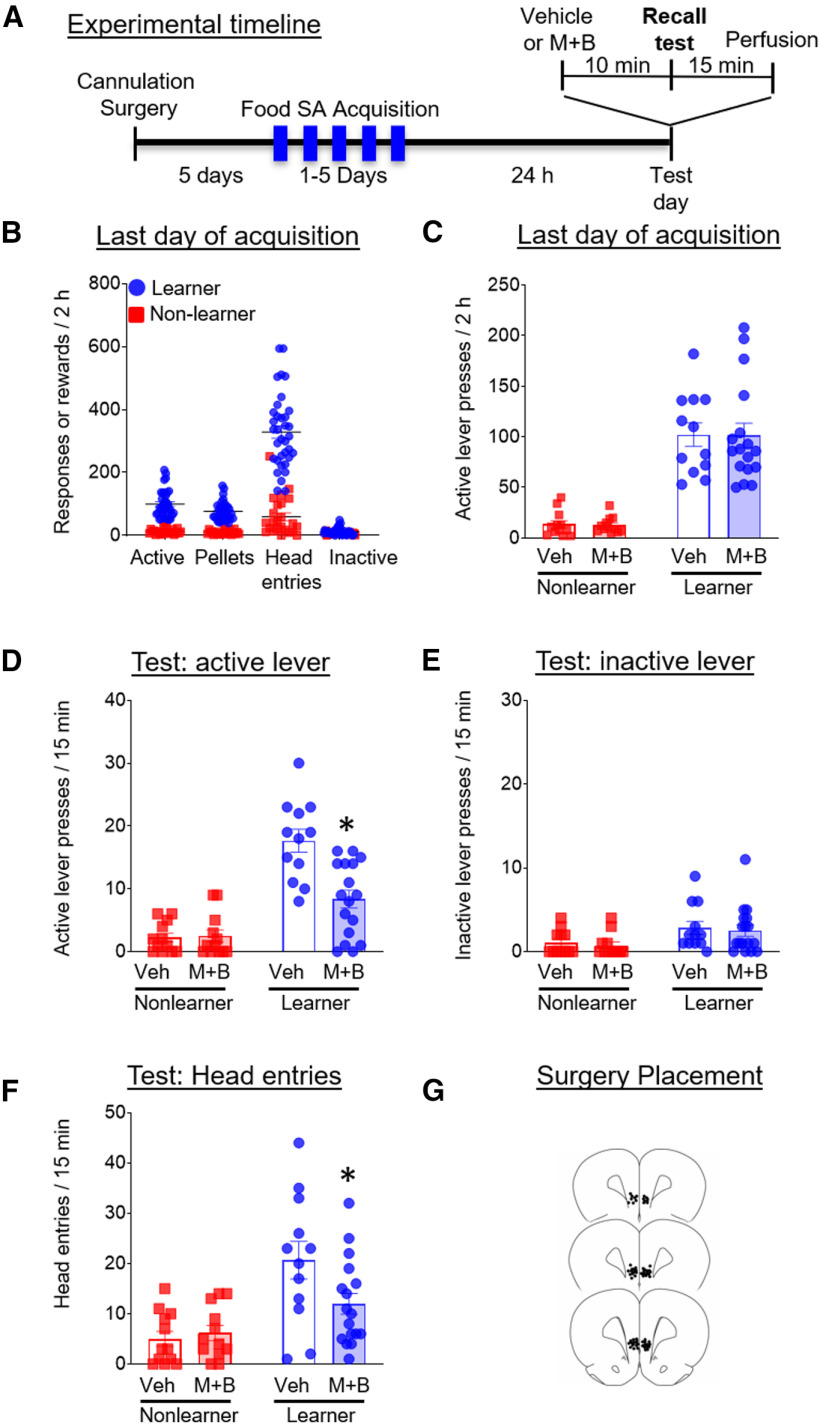
Experiment 2: effect of muscimol±baclofen inactivation of the IL during recall of food self-administration (SA). ***A***, Timeline showing the behavioral procedure. We performed cannulation surgeries 5 d before the start of food self-administration training. We trained rats to lever press for food for between 1 and 3 d in 2-h daily sessions, until rats self-segregated into learners and non-learners. On testing day, we infused muscimol+baclofen 10 min before the start of the 15-min testing session and perfused them after the test. ***B***, Number of active lever presses, pellets earned, head entries, and inactive lever presses during the last day of training. ***C***, Number of active lever presses during the last day of training for the groups that would subsequently receive vehicle or muscimol+baclofen on test day. ***D***, Number of active lever presses during the 15-min test day session for the learner and non-learner rats that received vehicle or muscimol+baclofen. ***E***, Number of inactive lever presses during the 15-min test day session for the learner and non-learner rats that received vehicle or muscimol+baclofen. ***F***, Number of head entries during the 15-min test day session for the learner and non-learner rats that received vehicle or muscimol+baclofen. ***G***, Images showing placement of cannulas into IL. Data are presented as mean ± SEM (*n* = 12–17 per group); *p < 0.05.

Here, we wanted to test the effect of region-wide inactivation of the IL on recall of food seeking after initial acquisition. The experimental timeline is shown in Figure 2*A*.

#### Behavioral responses during initial acquisition training

Figure 2*B* shows behavioral responses on the last day of self-administration. We used unpaired *t* tests to assess group differences in behavioral responses on the last day of self-administration training. By definition, learners demonstrated greater active lever presses, number of pellets earned, and head entries on the last day of self-administration training. We used a two-way ANOVA to assess group differences in mean active lever presses on the last day of self-administration training between preassigned drug conditions (vehicle vs muscimol+baclofen). We found a main effect of group (*F*_(1,49)_ = 84.6, *p* < 0.0001, η_p_^2^ = 0.64), no main effect of preassigned drug condition and no interaction (*p* > 0.05). A Holm–Sidak *post hoc* test showed that learners exhibited greater active lever presses compared to non-learners in both preassigned drug conditions (*p* < 0.05; Fig. 2*C*).

#### Behavioral responses during recall testing after region-wide inactivation of IL

We used a two-way ANOVA to assess group differences in active lever presses between learners and non-learners across drug conditions (vehicle, muscimol+baclofen). In these analyses, we include non-learners as a control matched in training experience to the learners, but lacking the key learning experience. This enables us to demonstrate that test responses of learners are not random. We found no significant differences in responding between male and female rats on test day and therefore combined their data for analysis. We found a main effect of group (*F*_(1,49)_ = 60.0, *p* < 0.0001, η_p_^2^ = 0.55), a significant main effect of drug (*F*_(1,49)_ = 10.9, *p* = 0.002, η_p_^2^ = 0.18) and a significant interaction between the variables (*F*_(1,49)_ = 12.1, *p* = 0.001, η_p_^2^ = 0.2). A Holm–Sidak *post hoc* test showed that muscimol+baclofen significantly decreased lever pressing in learners compared with vehicle-treated controls (*p* < 0.05; Fig. 2*D*). We used a two-way ANOVA to assess group differences in mean inactive lever presses between drug conditions (vehicle vs muscimol+baclofen). We found a main effect of group (*F*_(1,49)_ = 13.8, *p* < 0.01, η_p_^2^ = 0.22), but no main effect of drug, and no significant interaction between the variables; Fig. 2*E*). We used a two-way ANOVA to assess group differences in mean head entries between drug conditions (vehicle, muscimol+baclofen). We found a main effect of group (*F*_(1,49)_ = 20.3, *p* < 0.0001, η_p_^2^ = 0.3), but no main effect of drug, and a significant interaction between the variables (*F*_(1,49)_ = 4.3, *p* = 0.045, η_p_^2^ = 0.08). A Holm–Sidak *post hoc* test showed that muscimol+baclofen significantly decreased head entries in learners compared with vehicle-treated controls (*p* < 0.05; Fig. 2*F*). Together, these results suggest that muscimol+baclofen disrupted food-seeking behavior in learner rats.

### Experiment 3: effect of Daun02 inactivation of Fos-expressing neuronal ensembles in the IL during recall of food

**Figure 3.**
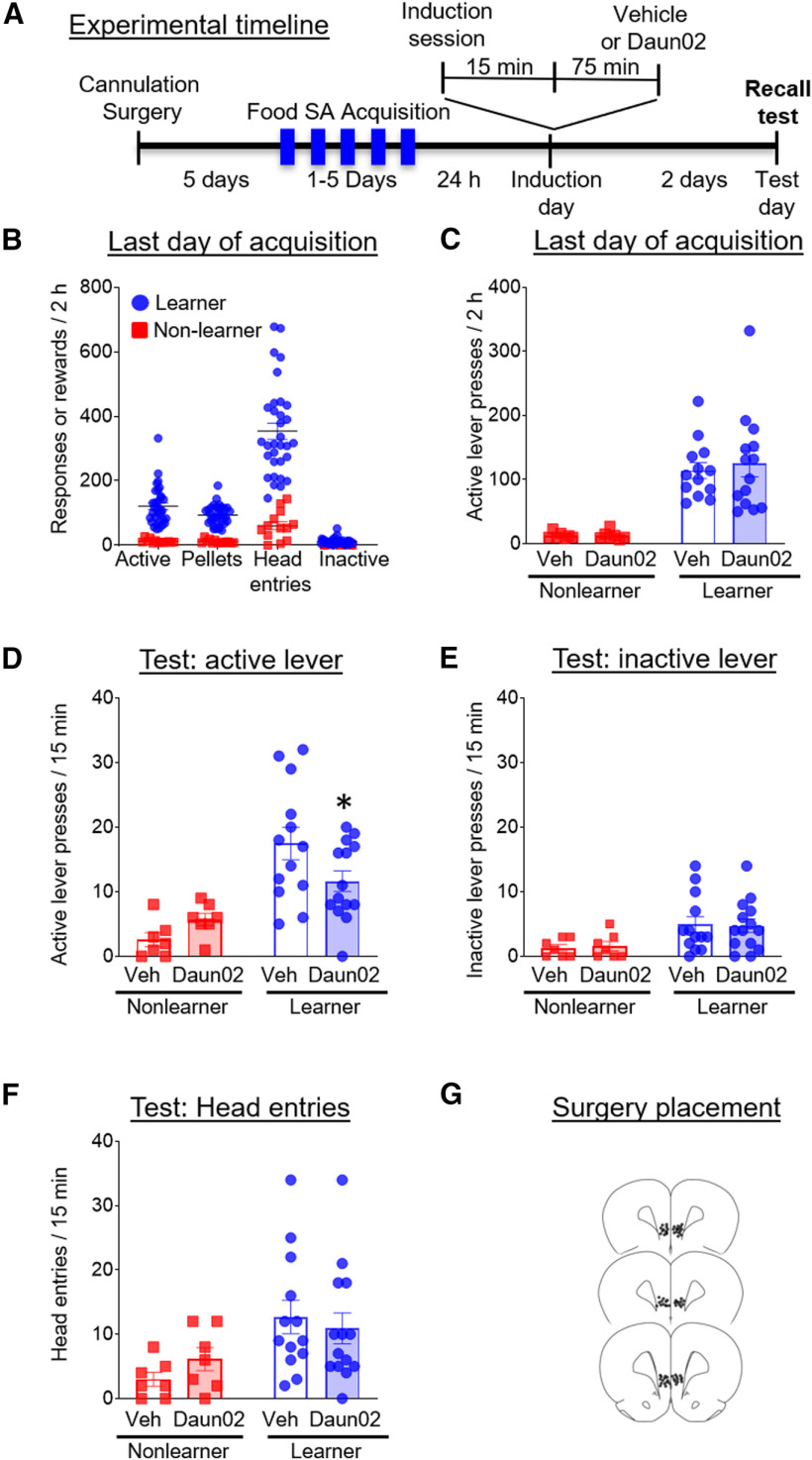
Experiment 3: effect of Daun02 inactivation of Fos-expressing neuronal ensembles in the IL during recall of food self-administration (SA). ***A***, Timeline showing the behavioral procedure. We performed cannulation surgeries 5 d before the start of food self-administration training. We trained rats to lever press for food for between 1 and 3 d in 2-h daily sessions, until rats self-segregated into learners and non-learners. On induction day, we exposed rats to an induction session for 15 min and infused vehicle or Daun02 75 min later. Two days later, on testing day, we tested rats for recall of the food self-administration memory in a test identical to the 15-min induction session. ***B***, Number of active lever presses, pellets earned, head entries, and inactive lever presses during the last day of training. ***C***, Number of active lever presses during the last day of training for the groups that would subsequently receive vehicle or Daun02 on induction day. ***D***, Number of active lever presses during the 15-min test day session for the learner and non-learner rats that received vehicle or Daun02. ***E***, Number of inactive lever presses during the 15-min test day session for the learner and non-learner rats that received vehicle or Daun02. ***F***, Number of head entries during the 15-min test day session for the learner and non-learner rats that received vehicle or Daun02. ***G***, Surgical placement of cannulas into IL. Data are presented as mean ± SEM (*n* = 7–14 per group); *p < 0.05.

We used the Daun02 inactivation procedure to determine whether Fos-expressing neuronal ensembles in the IL play a causal role in food acquisition. The experimental timeline is shown in Figure 3*A*.

#### Behavioral responses during initial acquisition training

By definition, learners exhibited a greater number of active lever presses, pellets earned, and head entries on the last day of self-administration (Fig. 3*B*). We used a two-way ANOVA to assess for preexisting group differences in mean number of active lever presses on the last day of acquisition. We found a main effect of group (*F*_(1,37)_ = 38.7, *p* < 0.0001, η_p_^2^ = 0.51), no main effect of drug, and no interaction. A Holm–Sidak *post hoc* test showed that learners exhibited greater responding compared with non-learners in the preassigned vehicle condition (*p* < 0.001) as well as in the preassigned Daun02 condition (*p* < 0.001; Fig. 3*C*).

#### Behavioral responses during recall testing after ensemble-specific inactivation of IL

We used a two-way ANOVA to assess group differences in mean active lever pressing between learners and non-learners across treatments (vehicle, Daun02). In these analyses, we include non-learners as a control matched in training experience to the learners, but lacking the key learning experience. This enables us to demonstrate that alterations in learners’ behavioral responses are not random. We found no significant differences in responding between male and female rats on test day and therefore combined their data for analysis. We found a main effect of group (*F*_(1,37)_ = 24.1, *p* < 0.001, η_p_^2^ = 0.39), no main effect of drug (*F*_(1,37)_ = 0.4, *p* = 0.53, η_p_^2^ = 0.01) and a significant interaction between the variables (*F*_(1,37)_ = 5.0, *p* = 0.03, η_p_^2^ = 0.11; Fig. 3*D*). A Holm–Sidak *post hoc* test showed that, in contrast to the pattern of effects observed among non-learners, Daun02-treated learners responded significantly less on test day compared with their vehicle-treated controls (*p* = 0.048). We used a two-way ANOVA to assess group differences in mean inactive lever presses between drug conditions (vehicle, Daun02). We found a main effect of group (*F*_(1,37)_ = 8.4, *p* = 0.006, η_p_^2^ = 0.187), but no main effect of drug, and no significant interaction between the variables; Fig. 3*E*). We used a two-way ANOVA to assess group differences in mean head entries between drug conditions (vehicle, Daun02). We found a main effect of group (*F*_(1,37)_ = 7.9, *p* = 0.008, η_p_^2^ = 0.18), but no main effect of drug, and no significant interaction between the variables (Fig. 3*F*). Thus, these results demonstrate Daun02 interfered with food-seeking behavior in learner rats.

## Discussion

Using a novel procedure to investigate acquisition of palatable food pellet self-administration, we found that wild-type rats self-segregated into those that rapidly learn food-seeking behavior (learners) and those that do not (non-learners). Compared with the non-learners, learners showed enhanced Fos expression within the PL and IL in response to stimuli previously associated with food self-administration, suggesting the involvement of these regions in food seeking. Congruent with previous experiments on food self-administration (Warren et al., 2016), we focused our experiments to investigate the role of the IL in acquisition of food-seeking. We tested the role of the IL in driving food-seeking behavior in learners using region-wide inactivation of the IL with muscimol+baclofen. We found a significant decrease in active lever pressing in learners infused with muscimol+baclofen compared with vehicle-treated controls, suggesting that the IL is necessary for food seeking after initial acquisition. We then employed Daun02 inactivation to specifically target only neurons that were strongly activated during a non-reinforced induction session. Selectively inactivating the Fos-expressing neuronal ensembles within the IL with Daun02 also decreased food seeking during the recall test in learners, compared with their vehicle-treated controls. Collectively, these data demonstrate a novel, functionally relevant role for Fos-expressing neuronal ensembles within the IL following the initial acquisition of food self-administration.

### The role of IL in natural reward seeking

The dorsal and ventral subregions of the mPFC are thought to have dissociable roles in mediating the execution and suppression of reward seeking, respectively. However, studies using region wide inactivation provide mixed support for this hypothesis (Rhodes and Killcross, 2004; Ishikawa et al., 2008a; Burgos-Robles et al., 2013; Sangha et al., 2014; Keistler et al., 2015). Others have hypothesized that the PL and IL differ in their response to goal-directed versus habitual responding. During early acquisition, reward-seeking is driven largely by goal-directed processes, thought to be governed by the PL, while overtrained behaviors engage habit-related processes, thought to involve the IL. Pharmacological inactivation studies using a reinforcer devaluation task in overtrained rats suggest a role for the IL in promoting habitual appetitive responding (Coutureau and Killcross, 2003; Killcross and Coutureau, 2003). Under these conditions, pharmacological inactivation of the IL did not influence food-seeking in undertrained rats, but blunted responding in overtrained rats. These findings contrast to the findings presented in this paper. The reason for this difference is unclear, but may involve the timing of the neurobiological manipulation. Pretraining lesions of the mPFC decrease the sensitivity of food-seeking behaviors to devaluation, but posttraining lesions do not (Ostlund and Balleine, 2005). An additional consideration is that rats that were considered undertrained in previous experiments still have substantially more training than in our model. While we endeavored to capture behavioral responding as soon as possible after the rat acquired the behavior, undertrained rats in previous studies were given multiple training sessions after acquisition and before testing.

Lastly, previous studies continued to reinforce the behavior during the test session. This could mask a recall-specific effect, as it is possible the rats could rapidly reacquire the behavior during testing, following a reinforced response. In any case, the results of the present study suggest an expanded role for the IL in appetitive seeking following initial acquisition.

Additionally, other studies suggest a role for the IL in modulating inhibitory associations in appetitive extinction following appetitive Pavlovian conditioning (Rhodes and Killcross, 2004). Experimental designs incorporating approach or aversive conditioning to assess contextually appropriate responses to a reinforcer or punishment should also be considered (Gentry and Roesch, 2018). Despite these varying ways of operationalizing appetitive behavior, the discrepant findings on the involvement of the dorsal versus ventral subregions of the mPFC may also be attributed to the use of region-wide manipulations rather than assessing behaviorally relevant neuronal subpopulations involved in different aspects of appetitive seeking. Here, our findings showed that both region-wide inactivation and ensemble-specific inactivation of the IL significantly decreased food seeking, which may highlight the relevance of the IL overall in appetitive seeking following initial learning.

Evidence from studies assessing single neuron recordings have found increased firing in the IL during cue-evoked appetitive approach responding, implicating the IL in reward seeking behavior (Burgos-Robles et al., 2013; Moorman and Aston-Jones, 2015; Gentry and Roesch, 2018). Additionally, neurons in both PL and IL regions fire in response to a rewarded stimulus and associated rewarded lever press as well as for non-rewarded stimuli associated with withheld behavioral responding (Moorman and Aston-Jones, 2015). Furthermore, single-unit recording of IL neurons show separate individual neurons activated by either pressing or abstaining from pressing under reinforcement or extinction conditions, respectively (Gentry and Roesch, 2018). These findings are consistent with previous studies demonstrating that distinct neuronal ensembles in the IL can mediate conditionally distinct behavioral responses — appetitive seeking and appetitive extinction (Suto et al., 2016; Warren et al., 2016).

Interestingly, muscimol+baclofen inactivation of the IL significantly reduced head entries, while Daun02 did not. One possible explanation is that the more targeted Daun02 manipulation disrupted test behavior that was restricted to the learned operant response, while muscimol+baclofen disrupted a less specific cluster of behaviors. Another potential explanation is that the head entries were lower in the Daun02 inactivation test and we are observing a floor effect. It is also possible that the lower level of Head entries make it difficult to observe a similar decrease.

In this study, although we found greater activation (indicated by Fos protein expression) within both PL and IL subregions in learners following a food-seeking recall test, we focused our subsequent experiments to specifically investigate the IL in mediating the earliest established food self-administration memories to expand on previous experimental findings and because we found a larger increase in Fos expression in the IL (Warren et al., 2016). Previous work has shown that PL-lesioned rats displayed delayed acquisition of operant responding for food and sucrose rewards (Corbit and Balleine, 2003), and other work has demonstrated a causal role of Fos expressing neuronal ensembles within the PL in mediating operant food seeking (Whitaker et al., 2017). Thus, neuronal ensembles within the PL should be further investigated in the acquisition of operant food-seeking.

### The role of IL projections in natural reward seeking

One of the main projections from the mPFC that has been investigated in reward seeking is to the nucleus accumbens (NAc; Sesack and Grace, 2010). Specifically, inactivation studies show that the PL projections to the core and the IL projections to the shell mediate initiation or extinction of reward seeking, respectively (LaLumiere et al., 2012). However, the IL projection to the NAc shell has also been shown to act as an inhibitory gate to mediate appetitive seeking during Pavlovian-to-instrumental transfer (Keistler et al., 2015). Additionally, in approach/avoidance studies, projections from the IL to the NAc have been suggested to drive choosing the most rewarding outcome while simultaneously inhibiting actions conflicting with that choice (Schwartz et al., 2017).

Furthermore, previous work has demonstrated a causal role of Fos-expressing neuronal ensemble projections from the IL to the NAc core in mediating cocaine seeking (Warren et al., 2019). The activation of this circuit may play a role in initial food seeking and should thus be further investigated with projection-specific methodologies. The involvement of Fos expressing neuronal ensembles along other projections such as BLA and PL to the NAc should also be considered as these pathways have been implicated in cue-evoked sucrose seeking (Ishikawa et al., 2008b).

A complex interplay of glutamatergic, GABAergic and dopaminergic signaling within the ventral tegmental area (VTA), mPFC, NAc, amygdala, and dorsal striatum have been shown to affect reward seeking. For example, GABAergic projection neurons from the central amygdala to the IL have been implicated in increasing operant responding for a sucrose reward (Seo et al., 2016). Glutamatergic projections from the mPFC and BLA to the NAc as well as dopaminergic projections from the VTA to the NAc have also been implicated in responding to reward predictive stimuli. Furthermore, dopamine excites NAc neurons via modulation of glutamate and GABA (Hjelmstad, 2004). Thus, cell type-specific circuit mapping of these neuronal ensembles should be considered. As distinct neuronal ensembles have been found within the same region to encode opposing forms of reward learning (Warren et al., 2017), characterizing their functional connectivity with other regions will be important to understanding how these ensembles mediate behavior, and may allow for manipulations that target specific memories without affecting others.

In conclusion, the current study found that greater neuronal activation within the IL was associated with acquisition of food self-administration. Both region-wide and neuronal-ensemble-specific inactivation of the IL decreased food seeking during a recall test. Considered together, these data suggest that IL neuronal ensembles are formed during the acquisition of food self-administration behavior and that these ensembles are necessary for the expression of food-seeking behavior. These experiments may shed light on the neurobiological underpinnings of food memories and motivation to acquire food.

## Synthesis

Reviewing Editor: Laura Bradfield, University of Technology Sydney Decisions are customarily a result of the Reviewing Editor and the peer reviewers coming together and discussing their recommendations until a consensus is reached. When revisions are invited, a fact-based synthesis statement explaining their decision and outlining what is needed to prepare a revision will be listed below. The following reviewer(s) agreed to reveal their identity: Arturo Zavala.

Dear Authors,

As you can see from the reviews below, both reviewers found your article to be well written and to contain some potentially interesting results. However, there are some concerns that need to be addressed. Most importantly, reviewer 2 suggests that the fact that you get a statistical difference with the Daun02 inactivation and not with region-wide inactivation could be a result of a relatively smaller sample size in the region-wide inactivation experiment, resulting in less power. At the very least it will be necessary to demonstrate (via report of a power analysis) that this is not the case. If the power analysis reveals that the current result is due to reduced power, then more animals will need to be added to the region-wide inactivation experiment. I believe that the result of this experiment - whether it leads to a significant difference between inactivated animals and controls or not - will be of interest either way.

I believe the novel contribution of your results is the demonstration that, despite the infralimbic cortex (IL) usually being thought of as having a role in extended or overtrained habitual behaviour, there is an ensemble of neurons within IL that is encoding and regulating early food-seeking. However, this point is not returned to in the discussion. It is interesting to note that in many of the studies that have demonstrated the role of IL in extended instrumental behaviour (e.g. Killcross & Couterau, 2003), region-wide inactivations did not affect undertrained instrumental actions. I think some consideration in the discussion of why here you have found evidence of neuronal ensembles within IL playing a role in early operant conditioning, whereas other studies have not, would help mitigate some of the reviewer concerns regarding novelty. It is worth noting (both for your current and for previous findings) that the failure to reject a null effect is not evidence that the effect does not exist, but rather represents the failure to find sufficient evidence that an effect exists.

The specific reviews are attached.

Reviewer #1

This is an excellent manuscript that is well designed and written,and it highlights the importance of examining underlying circuits within the infralimbic to understand food-seeking behavior. Importantly, it demonstrates that traditional approaches that inactive the infralimbic using muscimo+baclofen do not capture the contributions of this region to food-seeking behavior, and that it is necessary to use more mechanistic approaches, such as Daun02 inactivation, to highlight the involvement of this region. It would be beneficial to the reader to include a photomicrograph of the fos staining within the infralimbic. My only inquiry is with the authors statement that male and female rats were used in the study. Although it is unlikely that sex differences would emerge with such a small sample size, can the authors report what the sex distribution was across the groups? Were there an equal number of male or female rats that were learners vs nonlearners? If there is a natural separation in these groups by sex, can the authors comment, at the very least, the need to further examine sex differences.

Reviewer #2

This manuscript used an inactivation and Daun02 approach to examine how the infralimbic cortex (IL) controls early food self-administration. The authors found that Daun02-based lesions of IL cells that were activated by a food-seeking induction session reduced food seeking on the recall test day but only in “learners” - that is, those rats that had successfully learned the task. Overall, these are some interesting findings that suggest the development of ensembles in the IL that control food seeking. Generally, the manuscript is well written. My specific comments are below.

1) The difference between the muscimol + baclofen (M+B) experiment and the Daun02 study is interesting. However, looking at the data, it appears that the M+B experiment has a lower n than that of the Daun02 study. In particular, the vehicle-learner group in the M+B study has only 8 rats whereas the comparable group in the Daun02 study has 13. My own objective look at Figure 2D and 3D is that the magnitudes of the effect of M+B and Daun02 in the learner group are comparable with comparable variance. One possible explanation for the lack of a significant effect for the M+B learner experiment is that the M+B experiment is underpowered. The authors should do a power analysis of the comparison between the learners in that experiment to determine whether this might be the case. In addition, stating in the discussion unequivocally that there is no effect of M+B, in contrast to the effects observed with Daun02, seems too strong and should be constrained.

As a follow-up, it seems difficult to explain a lack of effect of the region-wide inactivation, as no competing or inhibitory ensemble should have been created by this point. Thus, it is difficult to imagine why taking the entire structure offline has no effect on behavior. It probably would have been better if the authors had run comparable numbers of rats in the M+B experiments as the Daun02 study so that they could more definitively discuss this issue (one way or the other).

2) A weakness is that the authors did not do more experiments that would have further defined the questions raised. For example, the paper would be considerably strengthened if the authors had attempted the same experiments in the prelimbic, particularly considering that they also found an increase in Fos expression there (Figure 1). In fact, the manuscript frames much of the Introduction on PL vs. IL, making the lack of PL data stand out even more. As it stands, the contribution of this work to the field, while interesting, is fairly small.

3) The authors state in the Methods that they also analyzed NAc for Fos expression, but there are no data presented for that and it is only ever mentioned again in the Discussion, where they note the “data is not shown”. Either the data should be shown or all mention of NAc should be removed.

3) In Figure 1C, the authors include “Pellets", but I believe this should be eliminated as nothing is there.

4) The authors refer to “Holms-Sidak” tests at various points, but it is “Holm-Sidak”.
